# 

**Published:** 1910-08-13

**Authors:** 


### BOOKS RECEIVED.

John Bale, Sons and Danielsson.
"The History of St. George's Hospital." By G. C. Peachey.

Hodder and Stoughton.
"Physiology, the Servant of Medicine." By A. D. Waller, M.D., etc.

Mills and Boon.
"Handbook for Nurses." By Sydney Welham.
"The Nursery Nurse's Companion." Honnor Morten.

Marlborough and Co.
"Esperanto Manual." By J. C. O'Connor, M.A.

G.E.R. Co.
"Tourist Guide to the Continent." By P. Lindley.

Oxford Medical Publication.
"System of Operative Surgery." By F. F. Burghard.

**The** recent Hospital Saturday procession at York again has proved both popular and successful, the total collections for the whole day amounting to £80. The hospital cot, the tableau representing a dirigible balloon, and the ambulance tableau were perhaps the most applauded cars in the procession.

### Gifts and Bequests.

Mr. Henry Dixon, of Cranford, has left £10,000 to the University of London upon trust to apply the income for scientific investigation in such manner as the authority of the said University may decide; and £2,000 to the West London Hospital for two beds.

Mr. William Chadwick, of Doncaster, J.P. for the West Riding, a life governor of the Doncaster and Leeds Infirmaries, has left £1,000 to the Doncaster Infirmary.

An anonymous donor has sent to the hon. treasurer of Leith Hospital £1,500 to endow a bed in memory of King Edward.

Among the latest contributions received at the Bank of England for King Edward's Hospital Fund for London are the following annual subscriptions:—Lord Iveagh, £500; Messrs. Coutts & Co., £250; Messrs. Glyn, Mills, Currie & Co., £250; Mr. Cecil H. Oliverson, £250; the Duke of Westminster, £100; Lord Barnard, £50; and Mr. Herbert Samuelson, £50.

The Ladies' Committee of the West London Hospital, Hammersmith, has received £201 from Mary Lady Ilchester, the proceeds of admission to Holland House Gardens.

The Royal Westminster Ophthalmic Hospital has received a donation of £20 from the Trustees of the Cholmondeley Charities.

Mr. Charles Kay, of Manchester, has left £150 each to the Ancoats Hospital and Ardwick and Ancoats Dispensary, and to the Northern Counties Hospital for Incurables.

Mrs. Frances Emily Palmer, of Tidenham, Glos, has left £500 and the proceeds of sale of her real estate at Lutterworth, Leicester, to the trustees of the "Feilding Palmer Cottage Hospital," Lutterworth.

Mr. Josiah Mander, of Overstone Park, Northampton, architect, bequeathed £400 to the Northampton Hospital.

Mrs. Ann Bowring, of Bradeley Moor, Wellington, Salop, who died on May 24, left £200 each to the Shrewsbury Eye and Ear Hospital, the Shrewsbury Infirmary, and the Port of Hull Society's East Coast Mission and Sailors' Orphan Home. She left her real estate in Springfield Place and Haygate Road, Wellington, as a site for the erection of a cottage hospital, and £3,400 for its erection, furnishing, and equipment. The balance of her residuary estate is to form an endowment fund for the hospital, which is to be known as the "John Crump Bowring Memorial Cottage Hospital."

### Jottings.

Lord Rothschild, treasurer of King Edward's Hospital Fund for London, has received the following letter :—

"Marlborough House, Pall Mall, S.W.,
August 1, 1910.

"My Lord,—I have it in command to acquaint your lordship of the King's gracious intention to augment his Majesty's yearly subscription to King Edward's Hospital Fund for London from £500 to £1,000, and I accordingly enclose a cheque for £500.

"The cheque is dated July 30 last, as the King is anxious that the date of the above augmentation should coincide with that of his Majesty's first visit to a London hospital since the accession.—I remain, my lord, your lordship's obedient servant, William Carington."

---

Princess Christian has become President of St. George's Hospital.

His Majesty the King has graciously consented to become patron of the German Hospital, Dalston, E.

Their Majesties the King and Queen have been graciously pleased to become patrons of the Royal Dental Hospital of London, Leicester Square, and the Italian Hospital.

Sir W. J. Collins, M.P., opened the new Edmonton Union Infirmary last month. The buildings, which occupy an extensive site adjoining the workhouse, can accommodate 400 patients, and cost less than £72,000.

*Black and White* of August 6 is of interest to hospital workers as it contains a number of drawings and photographs illustrating the visit of the King and Queen to the London Hospital, together with a special illustrated article on the work of the hospital.

Their Majesties the King and Queen inspected recently the Royal Hospital at Haslar, where they were received by Inspector-General T. Gimlette and conducted over the hospital, the medical and surgical officers and staff being at their posts in the wards of the hospital carrying out their respective duties.

The committee of the King Edward Memorial Fund in Birmingham have decided to give the commission for the marble statue of King Edward to Mr. Albert Toft, the cost not to exceed £2,000. The statue will be placed by the side of that of Queen Victoria, and therefore the Wright and Priestley statues will be removed to Chamberlain Square. The fund towards this and the erection of the Children's Hospital now amounts to over £13,000.

Mr. W. Pearson, Secretary of the Gravesend Hospital, Kent, sends us the following extract from a letter which has just come to hand from one of the supporters of the hospital : "When I went over the hospital I was proud to belong to it, as all was so well ordered, and this had such an effect that I enclose you £70 to make your total £1,000." The fund now stands at £1,010 as against £160 realised from a similar function last year. A marked success and a record! This year H. R. H. Princess Alexander of Teck received purses on behalf of the hospital, the customary fête not being held; the record established by Her Royal Highness' visit is a splendid proof of what personal service can do.

---

### TO HOSPITAL SECRETARIES AND OTHERS.

We invite contributions from any of our readers, and shall be glad to pay, according to length, for items of news for the institutional section (including appointments, resignations, gifts, etc.) which we may publish. All payments for manuscripts used are made as early as possible after the beginning of each quarter.

### The Hospital World.

The Local Government Board has sanctioned the hiring of a plot of land adjoining the infirmary at Merthyr for dealing with phthisical cases, and the Guardians have agreed to erect three shelters.

The Treasurer of St. Bartholomew's Hospital has received the following further contributions towards the Special Fund: Mr. Robert Fleming, £250; the Skinners' Company (second instalment of donation of £1,000), £200; " F. M. G.," £100; Mrs. Leighton, £50.

The Commmittee for the removal of King's College Hospital to South London has received a cheque for £1,000 from an anonymous donor for the purpose of naming a bed (to be called the "Inter Cruces" bed) in the new hospital at Denmark Hill.

Sir Henry Aubrey-Fletcher, Bart., of Ham Manor, Angmering, Sussex, late M.P. for Mid Sussex and for Horsham, a Groom-in-Waiting to Queen Victoria, left £100 each to the Sussex County Hospital, Brighton, the Worthing Hospital, and the Victoria Dispensary, Lewes.

